# Quantitation of methotrexate polyglutamates in human whole blood, erythrocytes and leukocytes collected via venepuncture and volumetric absorptive micro-sampling: a green LC–MS/MS-based method

**DOI:** 10.1007/s00216-022-04186-1

**Published:** 2022-07-07

**Authors:** Dala N. Daraghmeh, Mahin Moghaddami, Larisa Bobrovskaya, Susanna M. Proudman, Michael D. Wiese

**Affiliations:** 1 Centre for Pharmaceutical Innovation, UniSA: Clinical and Health Sciences, University of South Australia, GPO Box 2471, North Terrace, Adelaide, SA 5000 Australia; 2 Royal Adelaide Hospital and The University of Adelaide, Adelaide Medical School, Adelaide, SA Australia

**Keywords:** LC (HILIC)MS/MS, PBMC, RBC, Whole blood, VAMS, Rheumatoid arthritis

## Abstract

Low-dose methotrexate (MTX) plays a key role in treatment of rheumatoid arthritis. However, not all patients respond satisfactorily, and no therapeutic drug monitoring has been implemented in clinical practice, despite the fact that MTX therapy has now been available for decades. Analysis of individual intracellular MTX metabolites among rheumatoid arthritis (RA) patients is hampered by the low intracellular concentrations of MTX-PGs which require a highly sensitive method to quantify. Here, we present a rapid and highly sensitive LC (HILIC) MS/MS method with LLOQ 0.1 nM, 0.8 nmol/L for each metabolite of MTX-PG_1-5_ and MTX-PG_6-7_ respectively. Over a linear range of 0.1–100 nM, 0.8–100 nmol/L for each metabolite of MTX-PG_1-5_ and MTX-PG_6-7_, respectively, the inter- and intra- accuracy and precision were within 15% of the nominal value for all MTX metabolites. The presented assay was used to assess and compare MTX metabolite concentrations extracted from four different matrices: red blood cells, plasma, peripheral blood mononuclear cells, and whole blood that have been collected either using traditional venepuncture or volumetric absorptive micro-sampling (VAMS) sampling techniques. The presented method not only improves analyte coverage and sensitivity as compared to other published methods; it also improves the greenness.

**Graphical abstract:**

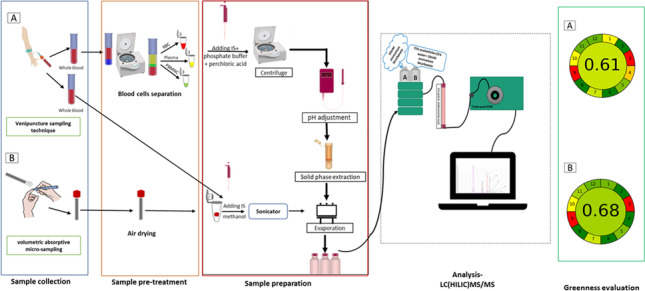

**Supplementary Information:**

The online version contains supplementary material available at 10.1007/s00216-022-04186-1.

## Introduction

**Fig. 1:**
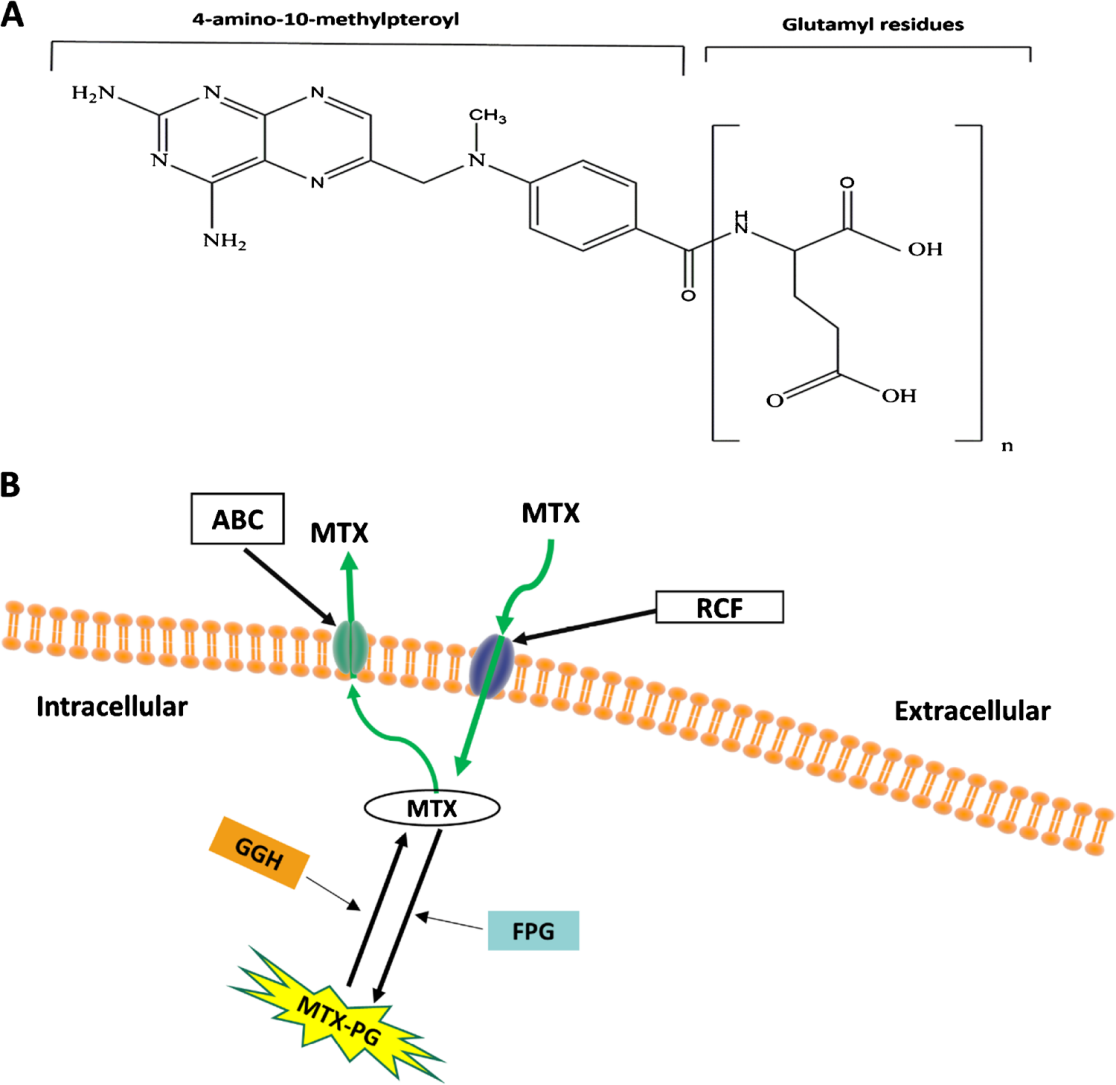
**A** Chemical structure of methotrexate, the glutamate group is indicated in brackets. **B** Methotrexate glutamination process. MTX: methotrexate, MTX-PG: MTX polyglutamate, RFC: reduced folate carrier, GGH: gamma-glutamyl hydrolase, ABC: ATP-binding cassette

Methotrexate (MTX) (4-amino-10-methylpteroylglutamic acid, Fig. 1) was designed in the 1940s and was initially used for the treatment of paediatric acute leukemia. The widespread use of MTX for the treatment of autoimmune inflammatory diseases such as rheumatoid arthritis (RA) occurred in the 1980s [1]. MTX dosing regimens used in the treatment of different diseases are variable; it is used at high intermittent doses for acute lymphoblastic leukemia, whereas it is used in low weekly doses in autoimmune diseases [2].

Following oral administration, MTX is absorbed from the small intestine by proton-coupled folate transporters, and approximately 10% is converted to a less active metabolite (i.e. 7-hydroxy-MTX) by hepatic aldehyde oxidase [3]. Once within the systemic circulation, MTX requires active transport via the reduced folate carrier (RFC) to enter target cells, upon which glutamic acid residues are attached to MTX by folyl-polyglutamate synthetase (FPG) to form the active metabolites, MTX polyglutamates (MTX-PGs). Gamma-glutamyl hydrolase (GGH) can reverse this polyglutamation process, and the resultant MTX can be transported out of the cell by efflux pumps belonging to the ATP-binding cassette family of membrane proteins [4] (Fig. 1). MTX contains a single glutamate residue in its parent chemical structure and up to 4 additional glutamic acid residues have been detected with low-dose MTX used in RA [4]. MTX-PGs are more potent inhibitors of target enzymes than MTX, and there are multiple proposed mechanisms of action in RA, including inhibition of dihydrofolate reductase, thymidylate synthase and methylenetetrahydrofolate reductase, which leads to depletion of purine and pyrimidine synthesis and ultimately interference of DNA synthesis [4].

Low-dose MTX has been the mainstay of RA treatment for decades, either as monotherapy or in combination with other disease-modifying anti-rheumatic drugs [5, 6], but there is significant inter- and intra-individual variability in clinical response, as up to about 40% of RA patients do not respond adequately, and many others suffer from adverse effects [7]. There has therefore been significant interest in the identification and clinical utility of biomarkers of MTX efficacy and toxicity [4], including measures of systemic MTX exposure. Measurement of plasma MTX concentration has recently been identified as a useful measure to determine patient adherence, with a recent study demonstrating that it can detect non-adherence to low-dose MTX in RA patients [8].

After oral or parenteral administration, plasma MTX concentration falls rapidly (plasma half-life ranges from 4.5 to 10 h) [9, 10], and MTX target enzymes are intracellular, so measuring plasma MTX concentration is unreliable for (directly) predicting response and toxicity. Intracellular MTX-PGs have a much longer half-life (weeks–months) [11] and have been proposed as a potential MTX response biomarker. Therapeutic drug monitoring (TDM) of intracellular MTX-PGs may have a role in optimising response to MTX, although data to support the utility of TDM in clinical practice is inconclusive so it has not been broadly implemented.

Quantifying individual intracellular MTX-PGs among RA patients is hampered by their low concentration, and as such a highly sensitive method is required. Only a few studies have quantified specific intracellular MTX-PGs in RA patients using LC–MS/MS [12–15] (Supplementary Table 1) or LC coupled with fluorescence detection [16]. The significance of very long MTX-PGs (i.e. MTX-PG_6-7_) in RA patients is not known, and it is unclear if they are not present, or if the methods developed to date have not been sufficiently sensitive to quantify them. The development of more sensitive methods may therefore allow MTX-PG_6-7_ to be quantified such that their importance in predicting MTX efficacy and/or toxicity can be better understood.

Quantification of intracellular RBC MTX-PG requires centrifugation of whole blood followed by removal of plasma and buffy coat, then preparation of washed RBC. This process is time-consuming and requires trained personnel and access to specialised laboratory facilities. Simplification of this procedure via bypassing these pre-treatment preparation steps by using whole blood-based samples instead of washed RBCs would improve the efficiency and accessibility of MTX-PG concentration monitoring. Furthermore, all methods that have quantified intracellular MTX-PGs from RA patients have used blood-based liquid samples which require venepuncture. This is costly and inconvenient for patients, and if drug concentration tests are requested at a clinical consultation, results are not available when the patient is with their clinician. There is therefore an increased interest in using micro-sampling techniques using dried blood spots (DBS) that potentially allow patients to collect their own samples, as they require only a small blood volume that can be obtained via a finger-prick. Samples are dried, which also overcomes the transport and storage requirements seen with those collected via venepuncture, but variability in haematocrit between patients can influence the accuracy of assays to quantify drug concentrations. Volumetric absorptive micro-sampling (VAMS) involves absorption of a fixed small volume of blood (i.e. 10 or 20 µL) following finger prick, regardless of the haematocrit, which overcomes the most common problems associated with DBS micro-sampling [17, 18]. If results with VAMS are similar to those observed from washed RBCs, the speed, convenience and simplicity of sample collection would offer a significant advantage over traditional sample collection techniques.

Blood is a mixture of RBCs (40–50%), white blood cells (WBCs) (~ 1% including peripheral blood mononuclear cells (PBMCs) and polymorphonuclear cells) and plasma (55%) [19]. PBMCs consist of three different cell types; lymphocytes (70–90%), monocytes (10–20%) and dendritic cells (1–2%) [20]. Intracellular concentrations of individual MTX-PG so far have only been measured in RBC. Since the therapeutic effects of MTX are mediated by intracellular enzymes and mainly involve alteration of leukocyte function, the site of action of MTX is more likely to be within WBCs (primarily lymphocytes) rather than RBC. The concentration within RBC is likely a surrogate for MTX-PG concentration at the primary site of drug action. RBCs are more abundant and have a longer half-life (i.e. about 120 days), so it is easier to access samples and measure the concentration of MTX-PGs within RBCs. However, the underlying biochemistry is different between RBC and PBMCs, and it has not been determined if the RBC concentration is an accurate predictor of PBMC concentration. As such, the concentration of MTX-PG within PBMCs may be quite different from that of RBCs, which may explain the relatively poor clinical utility of RBC MTX-PGs as a response biomarker, and PBMC MTX-PG concentration may be more predictive of MTX response.

This study aimed to develop and validate a highly sensitive and specific LC–MS/MS-based method and use it to quantify the concentration of MTX-PGs in different blood matrices: plasma, RBCs, PBMCs and whole blood that has been collected via either conventional venepuncture or finger-prick/VAMS techniques. Furthermore, given the awareness of more sustainable and environmentally friendly practices and the need to progress towards more economical and sustainable methods, the greenness of the developed method was evaluated against two recent greenness metrics; Green Analytical Procedure Index (GAPI) [21] and Analytical GREEnness Metric Approach and Software (AGREE) [22].

## Materials and methods

### Materials

MTX-PG_(1–7)_ were purchased as the ammonium salts from Schricks Laboratories (Bauma, Switzerland) and stable-isotope-labelled internal standards (13C5, 15 N), MTXPG_1-7_(+ 6), were purchased from Pepscan (Lelystad, The Netherlands). Ammonium bicarbonate (LC–MS Grade), ammonium hydroxide, monopotassium dihydrogen phosphate and perchloric acid were obtained from Sigma-Aldrich (Castle Hill, NSW, Australia). Density gradient medium (Lymphoprep™) was purchased from Axis-Shield (Oslo, Norway). LC–MS-grade methanol and acetonitrile (Honeywell, Burdick and Jackson™) were obtained from ChemSupply (Adelaide, Australia). Milli-Q water was obtained using a water purifier system (Sartorius, Arium®, Gӧttingen, Germany). Solid-phase extraction (SPE) columns (Strata-X-A Strong cationic SPE Tubes) were obtained from Phenomenex (Torrance, CA) and volumetric absorptive micro-sampling (VAMS) devices (20µL absorptive capacity) were purchased from Neoteryx (Torrance, CA).

### Preparation of standard and IS stock solutions

Stock standard solutions (100 μM) for each MTX metabolite (i.e. MTX-PG_1–7_) were prepared in 0.005 mM ammonium hydroxide in water, and then a 10 μM MTX_1-7_ mixed standard solution (i.e. containing 10 μM each of MTX_1-7_) was prepared. The mixed standard stock solutions were further diluted with Milli Q water to working solutions at concentrations of 1000 nmol/L, 100 nmol/L, 10 nmol/L and 1 nmol/L. Working Internal Standard (IS) stock solution (containing each stable-isotope-labelled MTX-PG_1-7_(+ 6)) was prepared from 10 μM stock solutions (in 0.005 mM ammonium hydroxide in water) and diluted with Milli Q water to a working solution that contained 100 nmol/L of each stable isotope MTX-PG_1–7_ standard. Stock solutions and working IS solutions were stored at − 20 °C prior to use. Phosphate buffers (0.1 M, pH = 2.7 and pH = 5.4) were freshly prepared on daily basis.

### Instrumentation

Liquid chromatography (LC) was performed with a Shimadzu HPLC apparatus (Kyoto, Japan) consisting of a gradient pump (model LC-30AD), an automatic injector (model SIL-30AC) and an on-line degasser (model DGU-20A_5R_). Analytes were detected with an AB Sciex® 6500^+^ triple quadrupole tandem mass spectrometer (Sciex, Framingham, MA) equipped with a turbo ion spray interface (API 6500 + MS/MS). Data acquisition and integration were carried out with Analyst software (Analyst 1.7, Sciex) linked directly to the LC–MS/MS.

### Chromatographic conditions and MS/MS detection

The separation of MTX and its metabolites was achieved with hydrophilic interaction liquid chromatography (HILIC) with a ZIC-pHILIC column (5 µm, 100 × 4.6 mm, Merck Millipore, Burlington, MA). Mobile phase A consisted of 75% acetonitrile in water with 10 mM ammonium bicarbonate and mobile phase B consisted of 10 mM ammonium bicarbonate in water. The following gradient was run at a constant flow rate of 0.4 mL/min. Initially, 100% of mobile phase A was maintained for 1 min, after which the percentage of mobile phase A was reduced to 40% via a linear gradient over 3 min. This was maintained for 2 min, before returning to 100% of mobile phase A over 1 min, which was maintained for 2 min prior to injection of the next sample. During analysis, samples were maintained at 15 °C and the column was maintained at 40 °C.

Mass spectrometry was performed in a positive ionisation multiple reaction monitoring (MRM) mode. Table 1 shows MRMs and the complete list of the compound-dependent parameters for each metabolite. Turbo gas temperature was set to 350 °C and sprayer voltage was set to 5500 V. Nitrogen gas was used and curtain gas was set to 20 psi, collision gas was set to 10 psi and ion source gas 1 and 2 were set to 50 psi and 60 psi respectively. Dwell time was set at 200 ms for each mass transition.

**Table 1:** Multiple reaction monitoring transitions and corresponding collision energy for methotrexate polyglutamate/s

Analyte	Precursor ion (*m/z*)	Product ion (*m/z*)	CE (V)	DP (V)	EP (V)	CXP (V)	Retention time (min)
Methotrexate	455.1	308.1	28	70	10	10	2.91
Methotrexate (+ 6)	460.9	308.1	26	60	8	9	
Methotrexate-PG_2_	584.1	308.1	38	80	10	14	3.52
Methotrexate PG_2_-(+ 6)	589.8	308.1	35	80	10	9	
Methotrexate-PG_3_	713.4	308.1	45	110	7	10	4.02
Methotrexate PG_3_-(+ 6)	718.9	308.1	46	110	10	16	
Methotrexate-PG_4_	842.2	308.1	55	63	9	16	4.30
Methotrexate PG_4_-(+ 6)	848.0	308.1	52	120	6	11	
Methotrexate-PG_5_	971.4	308.1	58	65	7	20	4.56
Methotrexate PG_5_-(+ 6)	976.9	308.1	58	145	10	9	
Methotrexate-PG_6_	550.3	308.1	29	110	9	21	4.73
Methotrexate PG_6_-(+ 6)	553.5	308.1	28	145	10	7	
Methotrexate-PG_7_	615.0	308.1	38	71	6	29	5.6
Methotrexate PG_7_-(+ 6)	618.3	308.1	30	145	10	8	

*CE* collision energy, *DP* declustering potential, *EP* entrance potential, *CXP* collision cell exit potential. (+ 6): stable-isotope-labelled internal standards (13C5, 15 N), MTXPG1-7(+ 6).

### Separation of plasma and red blood cells

Whole blood was centrifuged (2000 RCF at 4 °C for 10 min), which resulted in blood being separated into 3 layers: plasma at the top, RBC at the bottom and buffy coat in between. Plasma was carefully removed and stored at − 20 °C for later assay. Buffy coat was discarded and RBCs were removed, washed with PBS and centrifuged (2000 RCF at 4 °C for 10 min), then stored at − 80 °C.

### Separation of peripheral blood mononuclear cells

Whole blood was diluted with phosphate-buffered saline (PBS) in a 1:1 ratio, which was layered on top of Lymphoprep™ separation medium and centrifuged (600 RCF at 25 °C for 20 min), which resulted in blood being separated into 3 layers, top plasma-containing layer, PBMC interface layer and RBC at the bottom. The PBMC layer was washed with PBS; the number of cells was counted via microscopy and this was followed by centrifugation at 1200 rpm for 10 min at 4 °C. The supernatant was discarded and cell pellets were resuspended in 1 mL of PBS and stored at − 80 °C.

### Preparations of standards and quality control samples

Drug-free whole blood was collected from individuals not taking MTX and washed RBC prepared as above. Aliquots of drug-free RBCs (250 µL) were spiked with known amounts of MTX-PGs to yield standard solutions of known concentrations ranging from 0.1 to 100 nmol/L for MTX-PG_1-5_ and 0.8 to 100 nmol/L for MTX-PG_6-7_.

### Sample preparations

#### Red blood cell

Patient, standard and QC samples were all prepared identically. An aliquot of 250 µL packed RBC was lysed with 600 µL of Milli Q water, followed by the addition of 50 µL of IS solution. Protein was precipitated with 0.1 mL 30% perchloric acid and vortex mixed. Samples were centrifuged (3000 g for 10 min) and 0.8 mL of supernatant was removed, then 1.5 mL of 0.1 M of phosphate buffer (pH 5.4) was added and the sample was adjusted to pH 2.7. MTX-PGs were further purified by SPE using a Strata-X-A Strong cation exchange column. SPE columns were primed with 0.5 mL methanol followed by 0.5 mL of 0.1 M phosphate buffer (pH 2.7). The supernatant was then loaded onto the SPE column and allowed to flow through under gravity. Columns were sequentially washed with 0.5 mL of 0.1 M phosphate buffer (pH 2.7) and 0.5 mL methanol. Subsequently, samples were eluted with 0.5 mL of (5:95) 30% ammonium solution in methanol and evaporated to dryness under a stream of nitrogen. Samples were then reconstituted in 60 µL of mobile phase A and 8.5 µL was injected onto the LC column.

#### PBMC

Suspended PBMCs (500µL) were used in place of 250µL packed RBCs and water was not added. Subsequentially, the procedure used as described for RBC samples was followed.

#### Plasma

Instead of using 250µL packed RBCs, 250µL of plasma was used, then the same procedure as for RBC samples was then followed.

#### Whole blood sample preparation

Whole blood samples collected via VAMS.

VAMS devices were dipped in whole blood and held for 3 s until they became red, and devices were then left to dry for 3 h in air at room temperature. The VAMS were then placed into a clean Eppendorf tube and 2.5 µL of 1000 nmol/L IS solution was added, followed by 250µL of pure methanol. VAMS were then sonicated for 2 h and centrifuged at 12,000 rpm for 10 min. The supernatant was collected and evaporated to dryness under a stream of nitrogen. Samples were then reconstituted with 60µL of mobile phase A and 8.5µL was injected onto the LC column.

##### Whole blood samples collected via venepuncture

Whole blood (20 µL) was used without VAMS, and then 2.5 µL of 1000 nmol/L Internal standard solution was added, followed by 250µL of pure methanol. Samples were sonicated for 30 min and then centrifuged at 12,000 rpm for 10 min. The supernatant was collected and evaporated to dryness under a stream of nitrogen. Samples were reconstituted with 60µL of mobile phase A and 8.5µL was injected onto the LC column.

### Validation procedure

Prior to application on clinical samples, validation was performed according to FDA guidelines [23]. Selectivity, carry-over, lower limit of quantification (LLOQ), linearity, accuracy, matrix effect, recovery and stability were evaluated. Validation was performed with washed MTX-free RBC and whole blood that had been collected via venepuncture and VAMS. Given the scarcity and time-consuming nature of preparing blank PBMCs, validation was not performed using this matrix; instead, a comparison with calibration curves using RBC as the matrix was used [24].

### Validation procedure for samples in washed RBCs

### **Calibration curves**

A 7-point calibration curve over a concentration range of 0.1–100 nmol/L for MTX-PG_1-5_ and 0.8–100 for MTX-PG_6-7 _was prepared as per “Sample preparations-Red blood cells” and six replicates of each were run with quality control samples (i.e. low QC (QCL = 0.2 nmol/L), middle QC (QCM = 10 nmol/L) and high QC (QCH = 80 nmol/L for MTX-PG_1-5_ and QCL (2.4 nmol/L), QCM (15 nmol/L) and QCH (80 nmol/L) for MTX-PG_6-7_) on 4 different days. The analyte to IS ratio was calculated for each sample and the standard curve for each MTX metabolite was constructed using weighted (1/concentration) linear regression analysis using Prism software (San Diego, CA USA, www.graphpad.com).

### Accuracy and precision

Intra-day accuracy and precision for each MTX-PG were determined by analysing six replicates of the LLOQ and QC samples in each run. Accuracy was the relative difference between the calculated concentration and the spiked (known) concentration for each sample as per the equation below:

$$Accuracy=\frac{Calculated\;concentration-Known\;concentration}{Known\;concentration}\times100\%$$

Precision was defined as the coefficient of variation (CV%) and was calculated as the ratio of the standard deviation of the calculated concentration to the mean calculated concentration as per the equation below:

$$Precision\left(\%CV\right)=\frac{Standard\;deviation\;of\;the\;calculated\;concentration}{Mean\;calculated\;concentration}\times100\%$$

### Recovery

Recovery or the extraction efficiency was determined by comparing, on three separate days, the peak areas of all QC samples (i.e. QCL, QCM and QCH) that were prepared in washed RBCs with samples where the analytes were spiked with the same concentrations after the extraction procedure (i.e. blank RBCs underwent the extraction procedure and were subsequently spiked with MTX-PG and IS). The sample:IS area ratio was calculated and recovery was calculated by dividing the sample:IS where analyte was spiked before extraction with the sample:IS where analyte was added after extraction according to the equation below. Recovery was measured using six replicates.

$$Recovery=\frac{Ratio\;of\;ana\;nalyte\;to\;IS\;spiked\;before\;extraction}{Ratio\;of\;an\;analyte\;to\;IS\;spiked\;after\;extraction}\times100\%$$

### Matrix effect

Matrix effect was evaluated by comparing results obtained from QC samples (i.e. QCL, QCM and QCH) prepared in blank RBCs from 6 individuals who were not taking MTX where samples underwent the extraction procedure and were subsequently spiked with MTX-PG and IS into a blank matrix with results obtained from pure QC solutions (i.e. QC solutions prepared in water). The absolute matrix effect was calculated as the analyte:IS peak area of post-extraction spiked samples divided by the analyte:IS peak area from pure solutions.

$$Matrix\;effect=\frac{Ratio\;of\;an\;analyte\;to\;IS\;spiked\;post\;RB\;Cextraction}{Ratio\;of\;an\;analyte\;to\;IS\;extracted\;from\;pure\;solution}\times100\%$$

### Selectivity

The selectivity of the method was evaluated via analysis of blank RBCs from 6 individuals (3 females and 3 males) who were not taking MTX. Nonsignificant interference was defined as peak area responses at the expected retention times < 20% of the LLOQ response for all the analytes and < 5% of the IS response in the calibrators and QCs.

### Carry-over

Carry-over was assessed by regularly injecting a blank sample after the standard curve sample at the upper limit of quantification (i.e. 100 nM) and looking for any carry-over peaks. This was conducted 6 times during each of the 4 standard curve runs.

### Stability

Stability tests were evaluated using QCL, QCM and QCH samples that were prepared in washed RBCs as previously described. All samples were analysed in six replicates and results from stability test samples were compared with results from freshly prepared samples.

Freeze–thaw stability was examined by preparing QC samples and then extracting after 5 freeze–thaw cycles where samples were stored at − 80 °C and returned to room temperature prior to freezing again at − 80 °C. Bench top stability was tested by leaving samples at room temperature for 12 and 24 h and then spiking samples with IS. Autosampler stability was assessed by storing extracted samples for 24 and 48 h in the autosampler. Long-term stability was assessed after storage of samples at − 20 °C and − 80 °C for 1, 2 and 4 weeks and 7 months, after which samples were thawed, IS added and samples prepared as described above.

### Validation procedures used for samples in whole blood

Four calibration curves were prepared using whole blood samples collected via both venepuncture and VAMS using the method described above. The stability of MTX metabolites in whole blood collected via VAMS was evaluated by analysing the stored QC samples after storage for 2 weeks at 37 °C and room temperature (i.e. benchtop).

### Determination of MTX-PG concentration in PBMCs

The number of PBMCs in each sample was known and this was used to determine the volume of PBMCs in each sample by assuming that the volume of a single PBMC was 4 × 10^−13^L [25–27]. To determine the concentration of MTX-PGs in PBMCs, results were compared with a standard curve constructed from washed RBCs.

### Application

The developed assay was used to quantify MTX-PGs in washed RBCs, plasma, whole blood (collected via venepuncture) and capillary blood (collected via VAMS) in samples of whole blood and capillary blood that were collected at the same time from 8 RA patients who were receiving weekly MTX. Samples were prepared as described above. The four matrices (whole blood (collected via VAMS or venepuncture), washed RBC and plasma) were chosen to examine and compare if matrix composition and sample preparation affect total and individual MTX-PG concentrations. Ultimately, the aim of this comparison was to identify the simplest sample collection and preparation procedures that maintain accurate quantification of MTX-PGs.

Washed RBC and matched PBMC samples (i.e. prepared from the same blood sample) were collected from 5 RA patients who were receiving weekly MTX, and were used to examine the comparative MTX-PG_1-5_ distribution between each of the matrices. Samples were taken 1, 3, 6 and 9 weeks after initiation of weekly oral MTX.

### Greenness evaluations

The greenness of the developed LC–MS/MS methods and of other published methods [12, 14] was evaluated using GAPI and AGREE tools. Each of these tools uses the 12 principles of green analytical chemistry (GAC) [28] to assess the greenness of the analytical procedure from sample collection to quantification. GAPI is composed of five pentagrams (coloured red, yellow or green) to represent its environmental impact, where red represents high impact, yellow intermediate, and green low [21]. AGREE evaluates the environmental and occupational hazards associated with a particular procedure using all 12 GAC principles [28], and presents the results of the assessment in a clock-shaped graph in a quantitative manner on a 0–1 scale that contains an overall score [22].

### Statistical analysis

In the present study, the concentration of MTX-PG in RBC was determined as nmol/L of packed RBC, the concentration in whole blood collected via venepuncture was calculated as nmol/L of venous blood, samples collected via VAMS were presented as nmol/L of capillary blood and concentration in plasma was determined as nmol/L of plasma. The concentration in PBMCs was determined as nmol/L of PBMCs [24].

Data manipulation, graphical output and statistical analysis were performed in graph pad prism (version 8.3.0) and R software (version 4.1.1). Graph pad prism was used to construct the calibration curves and determine the concentration of each MTX-PG. R software was used for graphical output, using the ggplot2 [29], and the ggpubr package [30] was used to determine the Pearson correlation test to assess the magnitude of associations between the concentration extracted across different matrices. Student’s *t*-test was used to compare the difference of MTX-PG concentrations extracted from RBC versus PBMC samples. Statistical significance was accepted at a *p*-value < 0.05.

## Results and discussion

### Chromatography and mass spectrometry conditions

The developed LC–MS/MS method quantified MTX and its metabolites in RA patients in four different matrices: whole blood, washed RBCs, plasma and, for the first time, PBMC. Furthermore, the presented study applies a novel VAMS sampling technique to quantify MTX-PG in capillary blood and compares results with a conventional sampling approach.

**Fig. 2:**
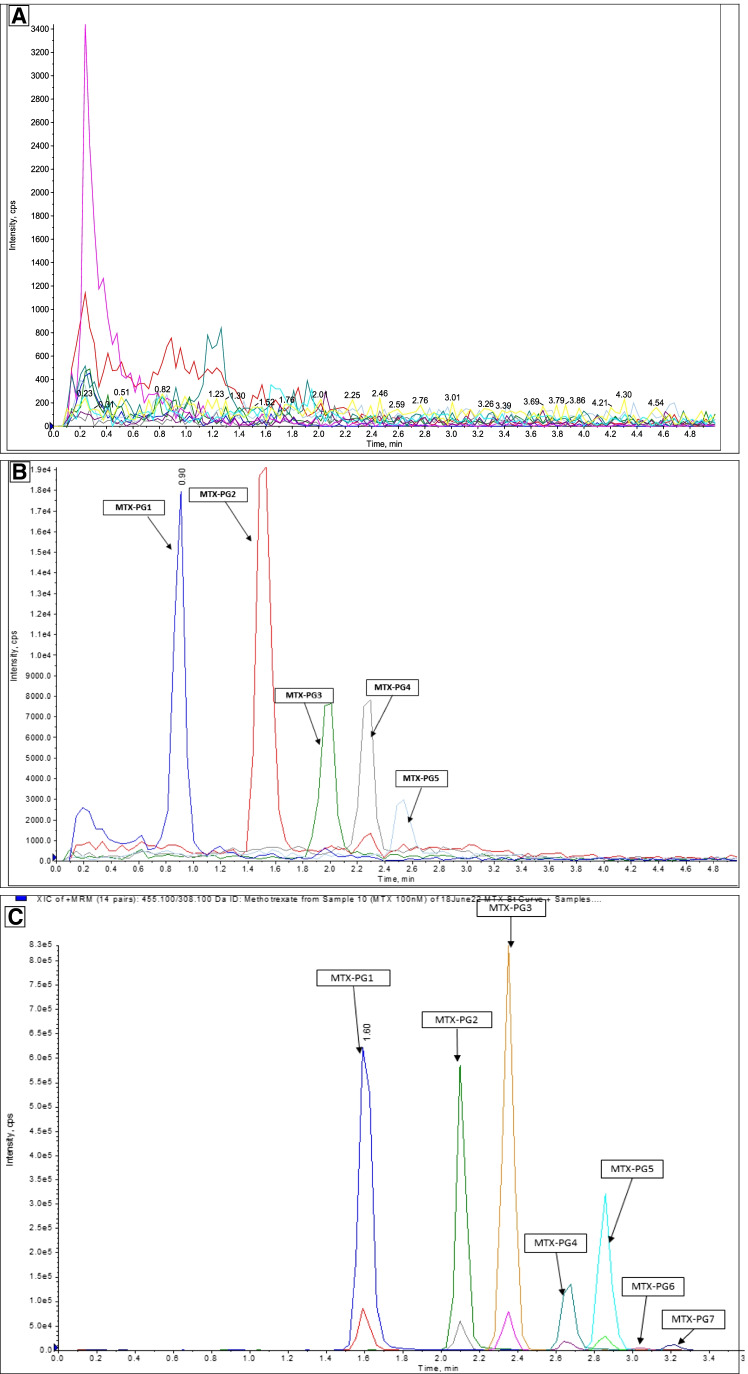
Representative chromatogram extracted from RBC; **A** blank matrix, **B** spiked RBC-0.1 nmol/L solution, **C** individual MTX-PG and its internal standards extracted from 100 nmol/L spiked RBCs

A representative chromatogram of each analyte and IS extracted from washed RBC is displayed in Fig. 2 and Supplementary Fig. 1. The retention time for each metabolite is summarized in Table 1. All previous LC–MS/MS methods used to quantify MTX-PG have used a reverse-phase high-performance liquid chromatography (RP-HPLC) [12–14] (Supplementary Table 1), whereas the presented method used HILIC, as it demonstrated better separation of the individual polar MTX-PG species (Fig. 2).

### Validation of LC–MS/MS assay

#### Calibration curve and lower limit of quantification

The calibration curves obtained for each of the seven MTX metabolites (MTX-PG_1-7_) were linear, and the inter-assay precision of the slope of each standard curve was ≤ 15% (Table 2). At the LLOQ (0.1 nmol/L and 0.8 nmol/L for MTX_1-5_ and MTX_6-7_ respectively), the imprecision was < 20% (Table 3).

**Table 2:** Linearity results of 4 calibration curves extracted from red blood cells

Analyte	*R*^2^ ± SD	Slope ± SD	CV	Intercept ± SD
MTX-PG_1_	0.999 ± 0.0011	0.141 ± 0.019	14%	0.277 ± 0.102
MTX-PG_2_	0.999 ± 0.0005	0.211 ± 0.025	12%	0.099 ± 0.052
MTX-PG_3_	0.999 ± 0.0008	0.239 ± 0.0126	5%	0.223 ± 0.114
MTX-PG_4_	0.999 ± 0.0004	0.098 ± 0.006	6%	0.113 ± 0.074
MTX-PG_5_	0.999 ± 0.0003	0.115 ± 0.007	6%	0.214 ± 0.076
MTX-PG_6_	0.994 ± 0.0043	0.0394 ± 0.001	3%	0.036 ± 0.022
MTX-PG_7_	0.994 ± 0.0030	0.0547 ± 0.0080	15%	0.0845 ± 0.079

Linearity based on 0.1–100 nM, 0.8–100 nmol/L calibration curve for MTX-PG_1-5_ and MTX-PG_6-7_ respectively. Slope represents the average of 4 linearity experiments in four different batches of pooled RBCs

**Table 3:** Assay performance of LOQ, QCL, QCM and QCH for each methotrexate metabolite prepared in washed red blood cells

Compound	Spiked concentration (nM)	Intra-assay (*n* = 4)		Inter-assay (*n* = 4)	
		Accuracy^*^ (range%)	Precision (range%)	Accuracy* (mean%)	Precision (%)
MTXPG-1	0.1 (LLOQ)	(− 14–12)	(0–6)	− 1	11
	0.2 (QCL)	(− 13–10)	(1–10)	1	10
	10 (QCM)	(− 7–6)	(6–8)	− 2	6
	80 (QCH)	(− 1–6)	(6–9)	3	3
MTXPG-2	0.1 (LLOQ)	(− 4–13)	(1–19)	4	9
	0.2 (QCL)	(1–9)	(5–12)	6	3
	10 (QCM)	(− 9–2)	(3–5)	− 3	5
	80 (QCH)	(0–4)	(1–5)	2	2
MTXPG-3	0.1 (LLOQ)	(− 7–19)	(2–16)	1	13
	0.2 (QCL)	(− 10–11)	(2–15)	0	9
	10 (QCM)	(− 3–7)	(7–11)	2	5
	80 (QCH)	(− 7–4)	(6–8)	0	5
MTXPG-4	0.1 (LLOQ)	(3–10)	(1–12)	8	3
	0.2 (QCL)	(− 11–8)	(2–14)	1	8
	10 (QCM)	(− 6–8)	(2–7)	− 1	6
	80 (QCH)	(− 1–5)	(1–4)	1	3
MTXPG-5	0.1 (LLOQ)	(− 4–10)	(1–5)	1	5
	0.2 (QCL)	(− 3–15)	(2–14)	2	11
	10 (QCM)	(− 6–0)	(2–7)	− 3	3
	80 (QCH)	(− 2–2)	(5–7)	1	3
MTXPG-6	0.8 (LLOQ)	(11–15)	(2–12)	13	2
	2.4 (QCL)	(− 8–9)	(3–10)	0	7
	15 (QCM)	(− 13–3)	(2–11)	− 5	8
	80 (QCH)	(− 4–3)	(5–8)	− 1	3
MTXPG-7	0.8 (LLOQ)	(− 14–15)	(1–6)	6	16
	2.4 (QCL)	(− 4–6)	(0–7)	2	4
	15 (QCM)	(− 14–2)	(2–9)	− 7	8
	80 (QCH)	(3–9)	(2–5)	5	3

*MTXPG* methotrexate polyglutamate, *LLOQ* lower limit of quantification, *QCL/M/H* quality control samples low/medium and high. *Accuracy was the relative difference between the calculated concentration and the spiked (known) concentration for each sample

Compared to all previously published methods where the reported LLOQ for individual MTX-PGs ranged from 1 to 5 nmol/L [12, 14, 16] (Supplementary Table 1), the sensitivity of the proposed method was improved for all MTX metabolites.

#### Accuracy and precision

Table 3 summarizes the inter- and intra-assay accuracy and precision for the LLOQ and QC samples, noting that they were within the defined acceptance criteria.

#### Recovery and matrix effect

The recovery of each MTX-PG was > 86% for all QC samples (Supplementary Table 3).

A comparison between chromatograms obtained from RBC samples obtained from six healthy volunteers who were not taking MTX after spiking blank RBC with the analytes and IS with pure QC solutions did not identify any matrix effect with the assay (Fig. 2).

#### Assay selectivity, specificity and carry-over

The developed method showed good selectivity for all analytes. Blank samples injected immediately after the upper standard curve sample (i.e. 100 nM) did not show noticeable peak areas, indicating no carry-over effect.

#### Stability studies

Spiked RBC samples stored at − 80 °C, − 20 °C and at room temperature (bench top) and extracted samples stored in the LC–MS/MS autosampler were stable for 7 months, 1 month, 24 h and 48 h respectively. Additionally, freeze–thaw cycle stability tests indicated that all MTX-PG metabolites in RBC were stable for five freeze–thaw cycles (Supplementary Tables 4, 5 and 6).

### Validation of methods using whole blood and VAMS

Methods developed using whole blood with and without VAMS adsorption demonstrated linearity between 0.1 and 100 nmol/L for MTX_1-5_ (Supplementary Tables 7 and 8). Supplementary Table 9 summarizes the inter- and intra-day accuracy and precision for QCs and LLOQ samples, and demonstrates that they were within the acceptance criteria (i.e. accuracy and precision < 15%). Furthermore, the ratio of the mean calculated concentration extracted from blood collected via VAMS compared to the calculated concentration for samples where MTX-PG was extracted from whole blood collected via venepuncture was all > 90% (Supplementary Table 8).

The results of stability studies demonstrated that MTX-PG_1-5_ in VAMS were stable at 37 °C and room temperature (benchtop) for 2 weeks (Supplementary Table 9), and benchtop stability evaluation showed that analytes were stable in whole blood samples at room temperature for 24 h (Supplementary Table 10).

### Application of the LC–MS assay: analysis of patient samples

#### RBC vs PBMC

**Fig. 3:**
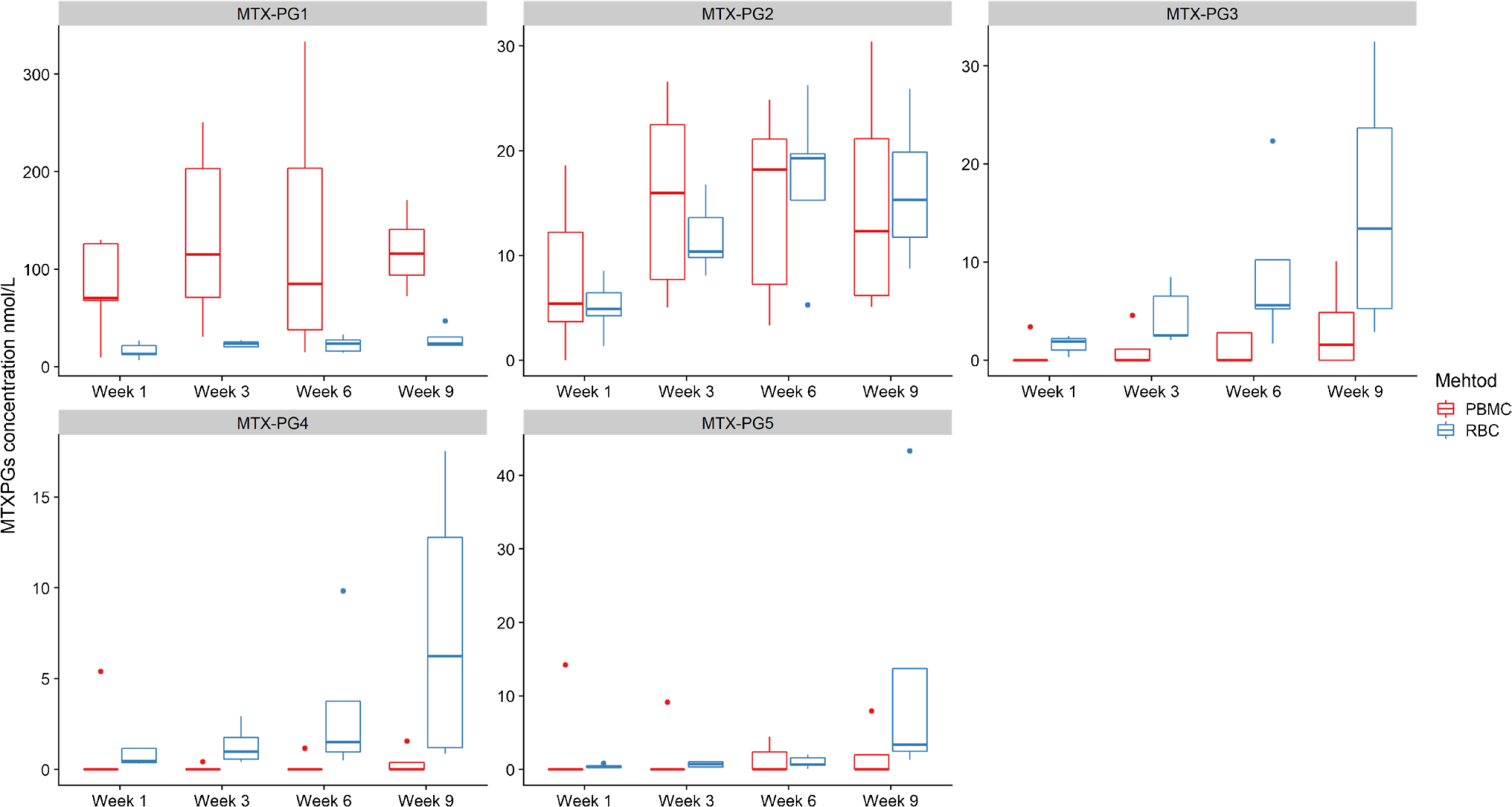
Log concentration profiles of individual MTX-PG metabolites extracted from peripheral blood mononuclear cells (PBMC) and red blood cells (RBC)

Figure 3 presents the MTX-PG_1-5_ concentration in RBC compared to PBMC at different time points. The distribution of MTX-PG was different between RBC and PBMC. Specifically, MTX-PG_1_ was the main metabolite present in PBMC at all times, with significantly higher concentrations compared to those present in RBCs (*P* < 0.01 at all times). MTX-PG_4_ and MTX-PG_5_ concentrations were lower in PBMCs compared to RBCs. Furthermore, total MTX-PG concentration was relatively low in RBC compared to the concentration determined in PBMC (Supplementary Fig. 2). There are several enzymes and transporters that likely contribute to the relative concentration of individual MTX-PGs in all cell types of the body, and differing expression of these may lead to different concentrations in different tissues of the body. Specifically, RFC is responsible for the uptake of MTX to the intracellular space, FPG is responsible for the addition of glutamic acid residues and GGH is responsible for the removal of glutamic acid residues. Mature RBCs lack RFC, whereas it is present in immature RBCs prior to their release into peripheral blood, and thus RBC MTX accumulation is more likely to occur in the bone marrow before RBC maturation [31, 32]. PBMCs at all stages have the RFC [33], and thus MTX accumulation is likely to occur throughout the PBMC life span. Furthermore, mature RBCs lack a cell nucleus and intracellular organelles, and therefore lack GGH and FPG enzymes, while this is not the case for PBMCs [34]. As such, given that GGH and FPG are able to constantly add and subtract glutamic acid residues, as the concentration of MTX-PGs in PBMCs is likely more dynamic than it is in RBCs. Furthermore, this explains the higher total concentrations in PBMCs shortly after treatment initiation compared with RBC as in RBC the accumulation occurs before RBCs are released into peripheral blood, while in PBMC it occurs throughout the entire lifespan. Given there are observed differences in the concentration of individual MTX-PG between RBC and PBMC, an investigation of the association between PBMC MTX-PG concentration and response to MTX is warranted.

#### Method comparison and analysis of patient samples

The four whole blood/RBC-based matrices used in this study were chosen to examine the differences in individual MTX-PG_1-5_ concentration according to matrix composition and sample preparation. Table 4 shows the total and individual concentration of MTX-PG_1-5_ extracted from washed RBC, whole blood, capillary blood adsorbed onto VAMS and plasma. Furthermore, the average concentration extracted from each individual MTX-PG_1-5_ in different blood matrices is presented in Table 4. The concentrations were similar to other values reported in the literature [6]. Using plasma samples, only MTX-PG_1_ and MTX-PG_2_ were detected in low concentrations, whereas five MTX metabolites (i.e. MTX-PG_1-5_) were quantified from all other matrices (Table 4). Thus, plasma MTX concentration is less likely to be useful as a marker for MTX efficacy. However, monitoring plasma MTX concentrations could help identify patient adherence, as this is highly variable and typically suboptimal with a reported rate ranging between 59% (underuse) to 107% (overuse) [35].

**Table 4:** Methotrexate metabolite concentration profiles extracted from RBC, plasma, WB collected via venepuncture and VAMS

Analyte	Sample	Concentration (nmol/L)				Ratio				
		WB	VAMS	RBC	Plasma	Plasma/WB	Plasma/VAMS	RBC/WB	WB/VAMS	RBC/VAMS
MTX-PG_1_	Patient 1	68.9	87.5	77.3	0.7			1.12	0.79	0.88
	Patient 2	42.2	43.9	39.5	0.9			0.94	0.96	0.90
	Patient 3	44. 3	50.9	43.1	4.5			0.97	0.87	0.85
	Patient 4	51.5	50.4	46.4	0.2			0.90	1.02	0.92
	Patient 5	28.3	25.5	22.9	1.1			0.80	1.11	0.90
	Patient 6	49.9	56.9	59.9	2.0			1.20	0.88	1.05
	Patient 7	60.9	64.3	70.6	0.3			1.16	0.95	1.10
	Patient 8	5.9	6.6	6.1				1.03	0.89	0.92
	Mean	43.9	48.3	45.7	1.4			1.01	0.93	0.94
MTX-PG_2_	Patient 1	19.0	22.0	22.4	0.4			1.18	0.86	1.02
	Patient 2	29.0	28.3	24.9	1.0			0.86	1.02	0.88
	Patient 3	34.3	34.0	30.0	3.1			0.87	1.01	0.88
	Patient 4	24.3	26.9	24.0	0.2			0.99	0.90	0.89
	Patient 5	9.3	8.9	7.6	1.0			0.82	1.05	0.85
	Patient 6	18.7	19.1	18.6	2.2			0.99	0.98	0.97
	Patient 7	22.2	22.2	19.5	0.1			0.88	1.02	0.90
	Patient 8	2.8	2.8	2.6	0.2			0.90	0.94	0.85
	Mean	20.0	20.5	18.7	1.0			0.94	0.97	0.91
MTX-PG_3_	Patient 1	31. 2	33.9	35.4				1.14	0.92	1.05
	Patient 2	91.5	105.9	105.9				1.16	0.86	1.00
	Patient 3	129.9	111.3	38.4				0.35	1.17	0.86
	Patient 4	87.1	96.0	85.0				0.98	0.91	0.89
	Patient 5	104.9	116.5	144.6				1.24	1.38	1.11
	Patient 6	13.7	13.2	14.4				1.05	1.04	1.09
	Patient 7	73.6	86.2	78.5				1.07	0.85	0.91
	Patient 8	10.0	13.1	10.6				1.06	0.77	0.81
	Mean	73.0	72.0	64.1				1.01	0.88	0.97
MTX-PG_4_	Patient 1	3.3	8.6	3.0				0.89	0.39	0.34
	Patient 2	9.1	9.7	7.8				0.85	0.94	0.80
	Patient 3	18.1	15.5	16.4				0.91	1.17	1.06
	Patient 4	18.1	17.5	20.2				1.12	1.03	1.15
	Patient 5	1.4	1.4	1.1				0.82	0.95	0.78
	Patient 6	26.2	29.2	28.3				1.08	0.90	0.97
	Patient 7	11.4	13.4	10.9				0.96	0.85	0.82
	Patient 8	0.7	0.6	0.8				1.02	1.17	1.19
	Mean	11.0	12.0	11.1				0.96	0.93	0.89
MTX-PG_5_	Patient 1	4.6	5.5	3.1				0.56	0.66	1.18
	Patient 2	2.7	2.8	0.9				0.32	0.96	0.33
	Patient 3	8.4	8.5	8.4				0.98	0.99	1.01
	Patient 4	8.2	11.7	4.5				0.39	0.70	1.43
	Patient 5	10.9	15.5	8.4				0.54	0.77	1.42
	Patient 6	1.7	2.6	1.4				2.03	3.15	1.55
	Patient 7	5.0	4.0	3.2				0.64	1.25	0.80
	Patient 8	12.9	14.2	10.9				0.77	0.84	1.10
	Mean	6.8	8.1	5.1				0.78	1.07	1.19
Total	Patient 1	127	157.5	141.2	1.1	0.009	0.007	1.11	0.81	0.90
	Patient 2	174.5	190.6	179	1.9	0.011	0.010	1.03	0.92	0.94
	Patient 3	235	220.2	136.3	7.6	0.032	0.035	0.58	1.07	0.62
	Patient 4	189.2	202.5	180.1	0.4	0.002	0.002	0.95	0.93	0.89
	Patient 5	154.8	167.8	184.6	2.1	0.014	0.013	1.19	0.92	1.10
	Patient 6	110.2	121	122.6	4.2	0.038	0.035	1.11	0.91	1.01
	Patient 7	173.1	190.1	182.7	0.4	0.002	0.002	1.06	0.91	0.96
	Patient 8	32.3	37.3	31	0.2	0.006	0.005	0.96	0.87	0.83
	Mean	149.5	160.9	144.7	2.2	0.014	0.014	1.00	0.92	0.91

*WBC* whole blood collected via venepuncture, *RBC* red blood cell, *VAMS* volumetric absorptive micro-sampling, *MTX-PG* methotrexate polyglutamate

Given the simplicity of sample preparation, determination of intracellular MTX-PG from whole blood (or capillary blood collected via VAMS) samples is advantageous over the need to prepare washed RBCs. However, it is likely that the inferred intracellular MTX-PG concentrations, particularly those of MTX-PG_1_ (parent MTX) from whole or capillary blood samples, will be artificially high in samples taken shortly after dose administration, as elimination of MTX from the plasma compartment is not yet complete. For this sampling strategy to be reliable, samples must be taken immediately prior to the next weekly dose being taken. This limitation must be acknowledged if whole (or capillary) blood is to be used as a sample matrix. The present study confirmed that most of the MTX-PG in whole blood was within the intracellular space, and only low amounts (< 3%) were in plasma. Furthermore, our study has demonstrated that total MTX-PG concentration calculated from washed RBC and whole blood samples (collected either by VAMS or venepuncture) were comparable (Table 4), with a mean ratio of calculated concentrations of 0.91–1.0 for RBC:whole blood, whole blood:VAMS and RBC:VAMS. One patient had a lower MTX-PG concentration in washed RBC concentration compared with concentrations calculated from samples collected via VAMS and venepuncture, although the same patient reported a high plasma MTX concentration (potentially indicating that the sample was not taken immediately before the next MTX dose was taken), which in part can help explain the lower concentration in washed RBCs.

**Fig. 4:**
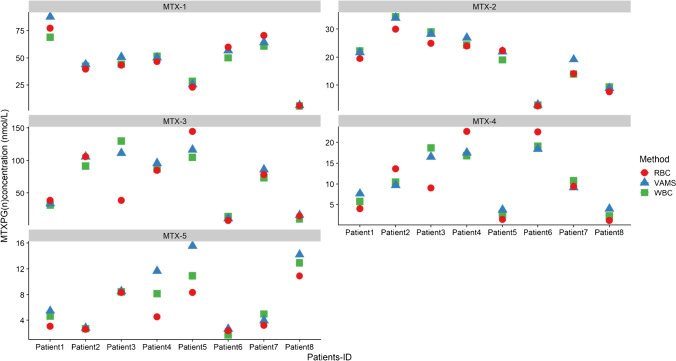
Concentration profiles for peripheral blood collected via VAMS versus venepuncture vs RBCs from rheumatoid arthritis patients receiving methotrexate

**Fig. 5:**
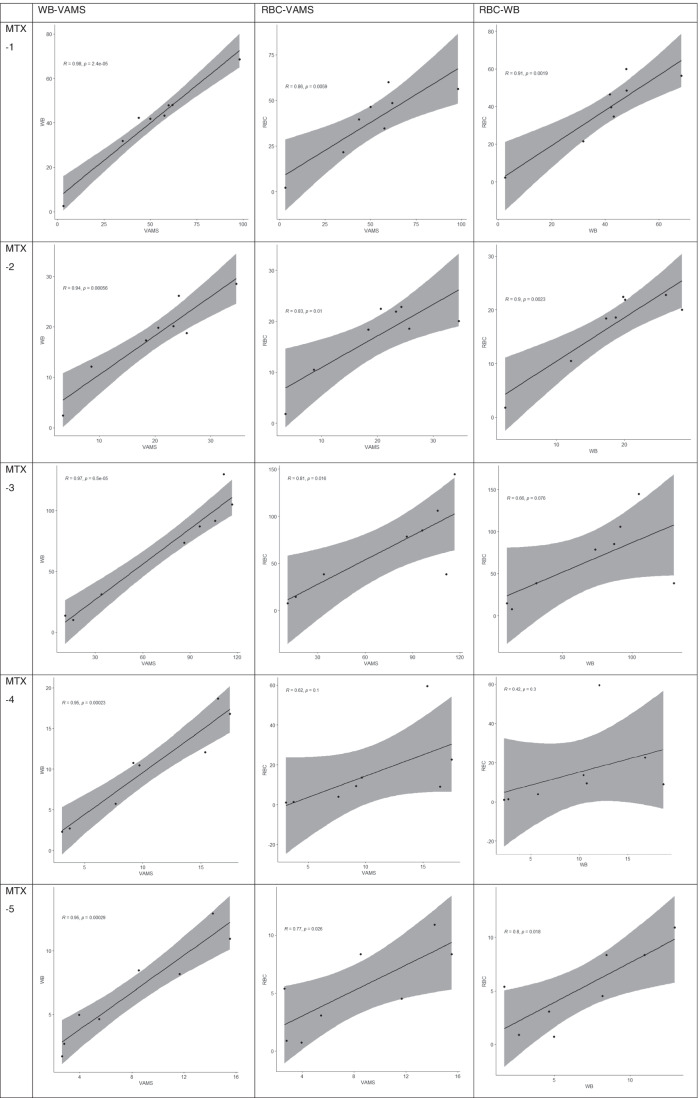
Correlation plots comparing the concentration of individual MTX-PGs metabolites extracted from red blood cells vs whole blood collected either via the conventional venepuncture or VAMS

The ratio of the calculated concentration of individual MTX metabolites extracted from whole blood, VAMS and washed RBCs ranged between 0.75 and 1.2 (Fig. 4). Although there was some variability, the correlation between the concentration calculated from samples derived from blood collected via VAMS and those extracted using venepuncture was high (R > 0.94) for all metabolites (Fig. 5). Although there are differences in the composition of samples collected via VAMS and venepuncture, as VAMS samples were collected from a finger prick which is considered capillary blood (i.e. a mixture of venous and arterial blood) mixed with small amounts of tissue whereas samples collected via venepuncture are venous blood, the calculated concentrations using the two matrices were very similar. Similarly, the comparison between MTX-PG concentrations determined from whole blood (collected by either venepuncture or VAMS) and washed RBC samples revealed a minimal difference in all but two patients (Table 4). Upon closer inspection, the correlation was higher for shorter chain MTX-PGs (MTX-PG_1-2_) whereas it was lower for the long-chain MTX-PGs (MTX-PG_3-5_) (Fig. 5). This may indicate that stability decreases with the increase in the number of additional glutamate residues and observed differences may be introduced during sample preparation. Dervieux et al. [16] have suggested that deproteinization by perchloric acid may lead to loss of long-chain MTX-PGs. Based on that, long-chain MTX-PGs are expected to be lower using the conventional deproteinization technique used in RBC (i.e. perchloric acid) compared with whole blood (collected either venepuncture or VAMS).

### Evaluation of greenness of the developed method

**Fig. 6:**
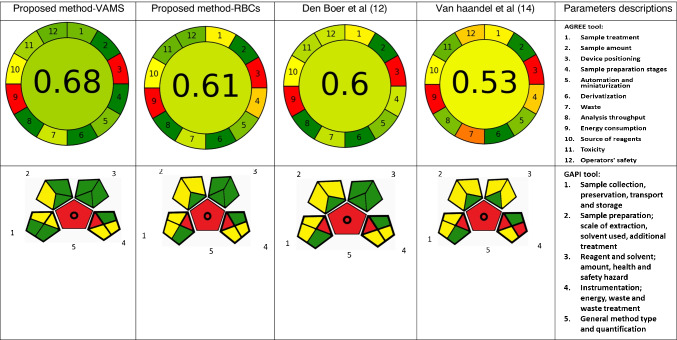
Comparison between the proposed methods “conventional venepuncture technique and the volumetric absorptive micro-sampling”, and other published methods

The GAPI pictogram demonstrates that the LC–MS/MS method where sampling was performed using the conventional venepuncture technique was green in 6 sectors, whereas using VAMS, 8 sectors were green. Only 2 and 3 sectors were red using the VAMS and conventional venepuncture sampling techniques respectively (Fig. 6). The AGREE assessment indicates that the greenness of the LC–MS/MS method was 0.61 for the conventional sampling technique and 0.68 for the VAMS technique. The detailed greenness evaluations using the two different tools are presented in Fig. 6. These results indicate that the VAMS technique is greener than the conventional venepuncture sampling technique since it uses less sample volume, avoids a sample pre-treatment step and reduces sample preparation steps and the amount of waste.

Additionally, the greenness of the proposed methods was compared with other published methods using GAPI and AGREE tools. All analytical methodologies reported 2 red sectors using the AGREE tool, and these were also red on the corresponding parts of GAPI, which results from energy consumption and off-line sampling with the LC–MS/MS instrument. The presented method (developed using washed RBC samples) was greener compared with the method reported by Van Haandel et al. [14] which presented an overall yellow shade with an AGREE score of 0.53 which was attributed to the higher number of sample preparation steps, longer run time and larger amount of waste. While a reported method by Boer et al. [12] showed a similar greenness profile using the GAPI tool (3 red sectors and 6 green) and a slightly different greenness profile using AGREE [12] (Fig. 6). Although very similar profiles were reported with den Boer et. al. [12], the method described within this manuscript resulted in improved sensitivity and coverage and similar greenness.

Finally, the validated methodology where sampling was performed using VAMS was assessed and compared to previously reported methods, and was simpler, superior in sensitivity and more ecofriendly.

## Conclusion

Typically, the first step of extracting small molecules from a biological sample such as blood is to determine the most suitable matrix that can be used, so choosing the most appropriate matrix for extraction is a significant step in bioanalytical method development. In this study, we used four different matrices to extract MTX and its polyglutamate metabolites to enable the most suitable matrix for measuring MTX-PGs accurately and precisely to be determined while considering patient, environmental and cost factors. Traditionally, washed RBCs have been used as a surrogate matrix for the main target such as PBMC, and here we found different distributions for MTX metabolites between these two matrices. The distribution of MTX-PGs in RBCs is different from the distribution in PBMCs, and therefore the reported RBC-MTX-PG concentrations may not be reflective of the concentration in PBMCs, which is assumed to be the site of action of MTX in RA. Investigation of response/toxicity relationships with MTX PBMC concentrations is therefore warranted to determine the utility of this method of sampling.

Sample preparation using peripheral whole blood to extract MTX metabolites without SPE or strong (perchloric) acid clean-up was achieved using a small amount of whole blood (i.e. 20 µL), which is more environmentally friendly and has fewer health risks. Additionally, MTX sample collection and preparation can be greatly simplified by using a VAMS sampling technique. This simplified, inexpensive method can readily be used for self-monitoring on-site testing as it enables home sampling which in turn helps increase awareness about medications and allow a shared-care approach and, thus has the potential to be beneficial in the implementation of TDM in clinical practice. Thus, using peripheral whole blood, collected using either venepuncture or VAMS, could save time while maintaining accuracy. However, samples must be taken immediately before the next dose of MTX is taken to ensure that calculated concentrations are not overestimated.

In summary, we have comprehensively demonstrated how MTX extraction can be achieved from different blood matrices, and how the MTX concentration calculated from different matrices relate to one another. Measuring MTX metabolite accumulation using various blood matrices has the potential to better understand MTX pharmacokinetics, and therefore modification of MTX dose regimens.

## Supplementary Information

Below is the link to the electronic supplementary material.

Supplementary file1 (DOCX 212 KB)

## Declarations

### Source of biological material

The blood samples were collected form RA patients who attended Central Adelaide Local Health Network rheumatology clinic.
