# Supplementary material for: Quantitation of methotrexate polyglutamates in human whole blood, erythrocytes and leukocytes collected via venepuncture and volumetric absorptive micro-sampling: a green LC–MS/MS-based method
Source: Anal Bioanal Chem. 2022 Jul 7;414(20):6029–46. doi: 10.1007/s00216-022-04186-1 (PMC9314280; doi:10.1007/s00216-022-04186-1)
Supplement: Supplementary file 1 — Supplementary file1 (DOCX 212 KB) [file 216_2022_4186_MOESM1_ESM.docx]

# Quantitation of methotrexate polyglutamates in human whole blood, erythrocytes and leukocytes collected via venepuncture and volumetric absorptive micro sampling: a green LC-MS/MS based method

# Authors

Dala N. Daraghmeh^1^, Mahin Moghaddami^1^, Larisa Bobrovskaya^1^, Susanna M Proudman^2^, Michael D. Wiese^1^

# Affiliation

1. Centre for Pharmaceutical Innovation, UniSA: Clinical and Health Sciences, University of South Australia, Adelaide, SA, Australia.
2. Royal Adelaide Hospital and The University of Adelaide, Adelaide Medical School, Adelaide, South Australia, Australia.

# Corresponding author

Address correspondence to Dala Daraghmeh, Centre for Pharmaceutical Innovation, UniSA: Clinical & Health Sciences, University of South Australia, GPO Box 2471, North Terrace, Adelaide SA 5000, Australia. Phone: +61 8 8302 1219, Email: [Dala.Daraghmeh@mymail.unisa.edu.au](mailto:Dala.Daraghmeh@mymail.unisa.edu.au), [**https://orcid.org/0000-0003-1933-5241**](https://orcid.org/0000-0003-1933-5241)**.**

# Supplementary tables and figures:

Supplementary Table 1. UPLC/HPLC–MS/MS method comparison for the detection of intracellular methotrexate metabolites in human health used in low doses.

| **Method** | **Matrix** | **Sample preparation** | **Separation mechanism** | **Column** | **Mobile phases** | **Analyte coverage** | **Time (min)** | **ESI** | **Calibration range** | **Ref** |
| --- | --- | --- | --- | --- | --- | --- | --- | --- | --- | --- |
| LC-MS/MS | WB  RBC  WBC  VAMS | PP with 30% perchloric acid+ SPE | HILIC | ZIC-pHILIC Merck (100×4.6 mm, 5µm) | A) 10 mM ammonium bicarbonate (B) 75% acetonitrile in water | MTXPG1-7 | 5-min | Positive | 0.1-100nM | Proposed method |
| LC-ESI-MS/MS | RBC | Protein was precipitated by adding 320μl cold 16 % perchloric acid to the diluted sample while vertexing and incubating on ice for 30 min, after which they were centrifuged for 15 min at 2,700×g | RP | Waters Acquity BEH C18 column (2.1 × 100 mm, 1.7μm) | (A) 10 mM ammonium bicarbonate adjusted to pH 10 with 25 % ammonium hydroxide and (B) methanol. | MTXPG1-5 | 6-min run time | Positive | 0.97–1,000 nM | [12] |
| LC-MS | RBC | Protein precipitation using perchloric acid followed by solid phase extraction using Oasis HLB SPE cartridges (30mg) | RP | Phenomenex synergy C18 (1.00 × 50mm, 4μm) | (A) 10 mM ammonium bicarbonate with 5mM DMHA (pH 7.5) and (B) acetonitrile with 5mM DMHA. | MTXPG1-7 | 20min | Positive | 0.5 to 100 nM | [14] |
| UPLC–ESI-MS/MS | RBC | Thermal extraction of erythrocyte lysate | RP | Waters BEH C18 column (1.00 × 50mm, 1.7μm | (A) 10 mM ammonium bicarbonate adjusted to pH 7.5 and (B) acetonitrile | MTXPG1-7 | 6 min | Positive | 1–1,000 nM | [13] |
| LC-MS | DBS | Protein precipitation using perchloric acid followed by solid phase extraction using MAX cartridges | RP | Atlantis T3-C18 column (3 µm, 2.1×150 mm) preceded by Atlantis guard column of matching chemistry | (A) 10 mM NH4HCO3 buffer adjusted to pH 7.5 using formic acid and (B) acetonitrile | MTXPG1-5  Total MTXPGs | 30 min | Positive | 5–400 nmol/L | [18] |
| LC-MS/MS | Serum | Protein precipitation using NaOH, and methanol followed by centrifugation | RP | Luna C18 (4.6×100mm, 3μm) | Mixture of 1% acetic acid–acetonitrile (88:12, v/v) | MTX | 6min | Positive | 10-1000 nM | [10] |
| LC-MS/MS | WB | freeze–thaw cycle and Protein precipitation with Trifluoroacetic acid and liquid-liquid extraction | RP | XB C18 (2.1× 100mm, 3μm) | Acetonitrile (1% formic acid)–20 mM ammonium formate solution (30:70, v/v) | MTX-PGs after conversion into MTX | 3min | Positive | 1-100 ng/mL | [15] |

RP: reverse phase, WB: whole blood, PBMC: peripheral blood mononuclear cells

|  | Blank RBC | LLOQ | ULOQ |
| --- | --- | --- | --- |
| MTX-1 |  |  |  |
| MTX-2 |  |  |  |
| MTX-3 |  |  |  |
| MTX-4 |  |  |  |
| MTX-5 |  |  |  |
| MTX-6 |  |  |  |
| MTX-7 |  |  |  |

Supplementary figure 1: Typical chromatogram of each analyte extracted from a blank RBC and at their respective lower limit of quantification (i.e., 0.1nmol/L for MTX-PG_1-5_ and 0.8nmol/L for MTX-PG_6-7_) and at upper limit of quantification (i.e., 100nmol/L for MTX-PG_1-7_)

Supplementary Table 2: Assay performance of methotrexate metabolites over the concentration range of 0.1-100nmol/L prepared in red blood cells

| Compound | Spiked concentration (nM/L) | Intra-assay (n=4) | | Inter-assay (n=4) | |
| --- | --- | --- | --- | --- | --- |
|  |  | Accuracy median (range%) | Precision median (range%) | Accuracy mean (%) | Precision  mean (%) |
| MTXPG-1 | 0.1 | (-14-12) % | (0-6) % | -1% | 11% |
|  | 0.5 | -12% (-14-12) % | 4% (1-12) % | -9% | 11% |
|  | 0.8 | 1% (-2-14) % | 5% (1-8) % | 11% | 13% |
|  | 15 | 6% (-7-12) % | 6% (4-7) % | 4% | 7% |
|  | 40 | -2% (-11-0) % | 6% (5-8) % | -3% | 5% |
|  | 100 | -3% (-8--2) % | 6% (3-10) % | -4% | 3% |
| MTXPG-2 | 0.1 | (-4-13) % | (1-19) % | 4% | 9% |
|  | 0.5 | 1% (-3-6) % | 3% (1-15) % | 1% | 3% |
|  | 0.8 | -2% (-6-14) % | 4% (2-12) % | 1% | 9% |
|  | 15 | -7% (-10--5) % | 6% (2-8) % | -7% | 2% |
|  | 40 | -1% (-8-2) % | 3% (3-5) % | -2% | 4% |
|  | 100 | -1% (-2-2) % | 3% (2-5) % | -1% | 2% |
| MTXPG-3 | 0.1 | (-7-19) % | (2-16) % | 0.90% | 12.47% |
|  | 0.5 | -3% (-8-9) % | 4% (3-15) % | -1% | 7% |
|  | 0.8 | 0% (-5-9) % | 6% (4-10) % | 1% | 7% |
|  | 15 | 0% (-4-4) % | 8% (4-9) % | 0% | 3% |
|  | 40 | -4% (-8-5) % | 6% (5-8) % | -3% | 6% |
|  | 100 | -1% (-4-3) % | 6% (5-12) % | -1% | 3% |
| MTXPG-4 | 0.1 | (3-10) % | (1-12) % | 7.74% | 3.30% |
|  | 0.5 | -3% (-11-6) % | 5% (3-8) % | -3% | 8% |
|  | 0.8 | 0% (-9-4) % | 4% (2-10) % | -1% | 6% |
|  | 15 | -3% (-7-6) % | 7% (2-14) % | -2% | 6% |
|  | 40 | -2% (-6-1) % | 3% (2-4) % | -2% | 3% |
|  | 100 | -1% (-3-2) % | 3% (2-6) % | 0% | 2% |
| MTXPG-5 | 0.1 | (-4-10) % | (1-5) % | 0.52% | 4.89% |
|  | 0.5 | -8% (-15-3) % | 8% (3-13) | -2% | 11% |
|  | 0.8 | 5% (-6-10) % | 3% (1-15) | 1% | 5% |
|  | 15 | 1% (-5-6) % | 5% (4-9) | 3% | 4% |
|  | 40 | -2% (-5-2) % | 7% (3-9) | -2% | 3% |
|  | 100 | 0% (-3-3) % | 6% (3-9) | 0% | 2% |

Supplementary Table 3: Extraction recovery test for samples; QCL, QCM, QCH

|  | Concentration | Recovery (%, Mean ± SD (n=3)) | CV% |
| --- | --- | --- | --- |
| MTX-1 | 0.2 | 107±0.038 | 4% |
|  | 10 | 90±0.139 | 15% |
|  | 80 | 86%±0.051 | 6% |
| MTX-2 | 0.2 | 127±0.048 | 4% |
|  | 10 | 93±0.045 | 5% |
|  | 80 | 100±0.119 | 12% |
| MTX-3 | 0.2 | 108±0.168 | 16% |
|  | 10 | 93.9±0.139 | 15% |
|  | 80 | 94.9±0.1077 | 11% |
| MTX-4 | 0.2 | 117±0.123 | 10% |
|  | 10 | 93.9±0.081 | 9% |
|  | 80 | 97.7±0.0970 | 10% |
| MTX-5 | 0.2 | 121±0.086 | 7% |
|  | 10 | 91.3±0.0840 | 9% |
|  | 80 | 98.6±0.136 | 14% |

Supplementary Table 4: bench top stability and auto-sampler

|  | Bench top | | | | | | | | | Auto-sampler stability | | | | | |
| --- | --- | --- | --- | --- | --- | --- | --- | --- | --- | --- | --- | --- | --- | --- | --- |
|  | 0 | | | 12hrs | | | 24hrs | | | 24hrs | | | 48hrs | | |
|  | % Accuracy (% CV) | | | | | | | | | | | | | | |
|  | QCL | QCM | QCH | QCL | QCM | QCH | QCL | QCM | QCH | QCL | QCM | QCH | QCL | QCM | QCH |
| MTX-PG1 | 8(14) | -15(11) | -10(15) | 1(6) | -14(1) | -9(13) | 9(10) | -15(8) | -9(12) | -11(4) | 12(13) | -7(7) | -10(10) | 11(2) | 11(5) |
| MTX-PG2 | 13(10) | 12(5) | -8(8) | -9(4) | 12(4) | 15(3) | 12(7) | -1(4) | -9(6) | 8(8) | 2(11) | -3(2) | 13(-2) | -7(5) | 2(0) |
| MTX-PG3 | 2(10) | -5(14) | 4(14) | -3(15) | -11(4) | 8(10) | 12(3) | -10(8) | -6(15) | 14(8) | 15(9) | 8(7) | 8(7) | 12(13) | 12(9) |
| MTX-PG4 | -2(1) | -7(5) | 4(2) | -1(7) | -14(1) | 13(7) | -2(8) | 5(6) | 2(13) | 15(10) | 5(5) | -5(3) | 4(14) | 14(3) | -3(3) |
| MTX-PG5 | -7(11) | 8(7) | 15(9) | 9(12) | 10(12) | 13(14) | 10(12) | 15(10) | 13(14) | 12(13) | -15(1) | 13(2) | 12(15) | -4(12) | -4(2) |

Supplementary Table 5: long term stability

|  | Long term stability at -20C | | | | | | | | | Long term stability at -80C | | | | | | | | |
| --- | --- | --- | --- | --- | --- | --- | --- | --- | --- | --- | --- | --- | --- | --- | --- | --- | --- | --- |
|  | 1 week | | | 2 weeks | | | 4 weeks | | | 2 weeks | | | 4 weeks | | | 7 months | | |
|  | % accuracy (% CV) | | | | | | | | | % accuracy (% CV) | | | | | | | | |
|  | QCL | QCM | QCH | QCL | QCM | QCH | QCL | QCM | QCH | QCL | QCM | QCH | QCL | QCM | QCH | QCL | QCM | QCH |
| MTX-PG1 | 10(6) | 9(11) | 8(14) | 7(6) | 9(12) | 12(10) | -10(8) | -10(6) | -6(12) | 15(2) | 2(10) | 7(9) | -9(12) | -13(14) | -14(8) | 14(7) | -5(13) | 3(14) |
| MTX-PG2 | 9(8) | 12(6) | 14(6) | 12(10) | -8(2) | -15(4) | -11(6) | -5(7) | -3(5) | 8(5) | -8(2) | 0(3) | -13(3) | -2(4) | -2(3) | -7(15) | -11(6) | -14(4) |
| MTX-PG3 | 13(3) | 14(13) | 10(14) | 3(8) | -14(3) | -15(4) | 9(7) | -1(6) | -9(5) | 12(5) | -15(2) | -13(7) | 12(5) | -13(9) | -10(10) | 12(9) | -11(5) | -14(5) |
| MTX-PG4 | -8(8) | 12(2) | 14(3) | 4(10) | -11(6) | -13(8) | 13(3) | 9(8) | 5(9) | -12(6) | -8(6) | -1(6) | 11(13) | 0(6) | 12(4) | 8(-8) | 12(7) | -1(3) |
| MTX-PG5 | 14(3) | 5(10) | 4(6) | 3(13) | 13(3) | 13(4) | 13(6) | -1(14) | -6(3) | 4(6) | 6(9) | -10(14) | 8(6) | -12(5) | -6(3) | 1(15) | 6(3) | 9(4) |

Supplementary Table 6: freeze thaw stability test

|  | Freeze-thaw stability | | | Freeze-thaw stability | | |
| --- | --- | --- | --- | --- | --- | --- |
|  | (Control) | | | (Test) | | |
|  | % Accuracy (% CV) | | | | | |
|  | QCL | QCM | QCH | QCL | QCM | QCH |
| MTX-PG1 | 0(12) | 15(2) | 9(14) | 2(8) | -7(15) | 7(12) |
| MTX-PG2 | 9(1) | -15(8) | 4(7) | 7(10) | -14(2) | 4(4) |
| MTX-PG3 | 13(7) | -15(8) | 4(7) | 9(6) | -14(7) | 8(7) |
| MTX-PG4 | -9(4) | -14(6) | 11(2) | -14(4) | -14(4) | 10(3) |
| MTX-PG5 | 0(7) | -13(8) | 3(6) | 3(6) | -4(7) | -3(4) |

Supplementary Table 7: linearity results of 4 calibration curves extracted from peripheral blood collected via VAMS versus venepuncture

| Analyte | Slope ±SD | Intercept ±SD |
| --- | --- | --- |
| VAMS | | |
| MTX-PG_1_ | 0.0108±0.00089 | 0.0663±0.0287 |
| MTX-PG_2_ | 0.0172±0.001891 | 0.0478±0.0123 |
| MTX-PG_3_ | 0.0227±0.003 | 0.0989±0.0357 |
| MTX-PG_4_ | 0.0035±0.0001 | 0.0384±0.00276 |
| MTX-PG_5_ | 0.0026±0.00043 | 0.0357±0.0081 |
| Whole blood cells | | |
| MTX-PG_1_ | 0.0272±0.00997 | 0.0687±0.01861 |
| MTX-PG_2_ | 0.0687±0.0264 | 0.0821±0.0316 |
| MTX-PG_3_ | 0.0285±0.007 | 0.1001±0.0573 |
| MTX-PG_4_ | 0.0167±0.004 | 0.0801±0.0725 |
| MTX-PG_5_ | 0.0087±0.003 | 0.0357±0.0048 |

Supplementary Table 8: Assay performance of LLOQ, QCL, QCM and QCH for each methotrexate metabolites extracted from blood cells that were collected via venepuncture or VAMS

| Analyte | Concentration (ng/mL) | Whole blood collected via venepuncture | | | | VAMS | | | | VAMS / venepuncture | |
| --- | --- | --- | --- | --- | --- | --- | --- | --- | --- | --- | --- |
|  |  | Intra-day | | Inter-day | | Intra-day | | Inter-day | | Inter-day | Intra-day |
|  |  | Accuracy (%) | Precision (%) | Accuracy (%) | Precision (%) | Accuracy (%) | Precision (%) | Accuracy (%) | Precision (%) |  |  |
| MTX-PG_1_ | LLOQ | 2% | 5% | 2% | 8% | 14% | 0% | 12% | 5% | 90% | 88% |
|  | QCL | -5% | 10% | -2% | 10% | 1% | 1% | 1% | 4% | 97% | 94% |
|  | QCM | 1% | 11% | 1% | 3% | -5% | 4% | -4% | 10% | 105% | 106% |
|  | QCH | -5% | 11% | -5% | 9% | 5% | 6% | 4% | 3% | 91% | 90% |
| MTX-PG_2_ | LLOQ | -7% | 4% | -8% | 8% | -5% | 1% | -5% | 8% | 97% | 98% |
|  | QCL | -3% | 3% | -4% | 9% | 1% | 1% | 1% | 7% | 95% | 96% |
|  | QCM | 6% | 3% | 4% | 6% | -3% | 1% | -1% | 11% | 105% | 109% |
|  | QCH | -5% | 5% | -5% | 10% | 2% | 2% | 2% | 5% | 93% | 93% |
| MTX-PG_3_ | LLOQ | 1% | 9% | -1% | 4% | 9% | 2% | 4% | 17% | 95% | 92% |
|  | QCL | -7% | 4% | -2% | 9% | -4% | 2% | -2% | 13% | 100% | 97% |
|  | QCM | 1% | 5% | 2% | 2% | -2% | 4% | -1% | 12% | 103% | 103% |
|  | QCH | -6% | 6% | -5% | 9% | 2% | 4% | 3% | 6% | 92% | 92% |
| MTX-PG_4_ | LLOQ | -5% | 4% | -3% | 13% | -13% | 1% | -7% | 14% | 104% | 108% |
|  | QCL | 10% | 3% | 4% | 11% | 4% | 0% | 5% | 8% | 99% | 106% |
|  | QCM | 3% | 7% | 4% | 3% | 2% | 4% | 2% | 7% | 102% | 101% |
|  | QCH | -5% | 3% | -5% | 13% | 5% | 4% | 3% | 5% | 92% | 90% |
| MTX-PG_5_ | LLOQ | -2% | 4% | 2% | 6% | 0% | 1% | 1% | 10% | 101% | 98% |
|  | QCL | -6% | 2% | -7% | 2% | 0% | 0% | 0% | 10% | 93% | 94% |
|  | QCM | 3% | 3% | 6% | 3% | 2% | 1% | 3% | 6% | 103% | 101% |
|  | QCH | -5% | 3% | -3% | 8% | 3% | 5% | 2% | 5% | 95% | 92% |

Supplementary Table 9: Stability of MTX metabolites extracted from peripheral blood cells collected via VAMS

|  | Stability tests- VAMS | | | | | | | | |
| --- | --- | --- | --- | --- | --- | --- | --- | --- | --- |
|  | 0 | | | 2 weeks- Bench top | | | 2 weeks at 37^o^C | | |
|  | % Accuracy (% CV) | | | | | | | | |
|  | LQC | MQC | HQC | LQC | MQC | HQC | LQC | MQC | HQC |
| MTX-PG1 | 2(1) | -9(4) | 4(6) | 10(12) | 10(14) | 7(14) | 10(15) | -13(14) | 6(9) |
| MTX-PG2 | -8(3) | -3(5) | 2(2) | 7(7) | 8(13) | 14(12) | 8(8) | 11(6) | 5(6) |
| MTX-PG3 | 0(3) | -8(4) | 3(4) | 9(11) | 14(10) | 11(7) | 10(14) | 15(7) | 10(10) |
| MTX-PG4 | 10(1) | -3(5) | 3(6) | 10(13) | 11(13) | -8(3) | -1(12) | -4(14) | 15(4) |
| MTX-PG5 | -11(1) | 4(1) | 2(5) | 4(14) | 13(14) | 9(7) | -6(8) | 14(8) | 13(7) |

Supplementary Table 10: Stability of MTX metabolites extracted from peripheral blood cells collected via venepuncture

|  | Stability tests- Whole blood collected via venepuncture | | | | | | | |
| --- | --- | --- | --- | --- | --- | --- | --- | --- |
|  | 24hrs-Bench top^*^ | | | | 24hrs-Bench top | | | |
|  | % Accuracy (% CV) | | | | | | | |
|  | LLOQ | QCL | QCM | QCH | LLOQ | QCL | QCM | QCH |
| MTX-PG1 | 10(16) | 14(9) | -7(9) | 10(12) | 9(17) | -12(8) | -7(12) | 10(15) |
| MTX-PG2 | 15(3) | 14(5) | -13(6) | 11(7) | 10(8) | 1(7) | 10(11) | 14(6) |
| MTX-PG3 | 7(3) | -5(1) | 3(4) | 15(1) | 12(11) | -8(9) | 3(4) | 0(4) |
| MTX-PG4 | -13(5) | -12(3) | 13(5) | 0(3) | 11(8) | -5(5) | 12(5) | -10(6) |
| MTX-PG5 | 12(14) | 13(2) | 14(5) | -13(6) | 16(15) | 10(15) | 14(15) | 14(3) |

*: prepared using the same method used to prepare RBC samples


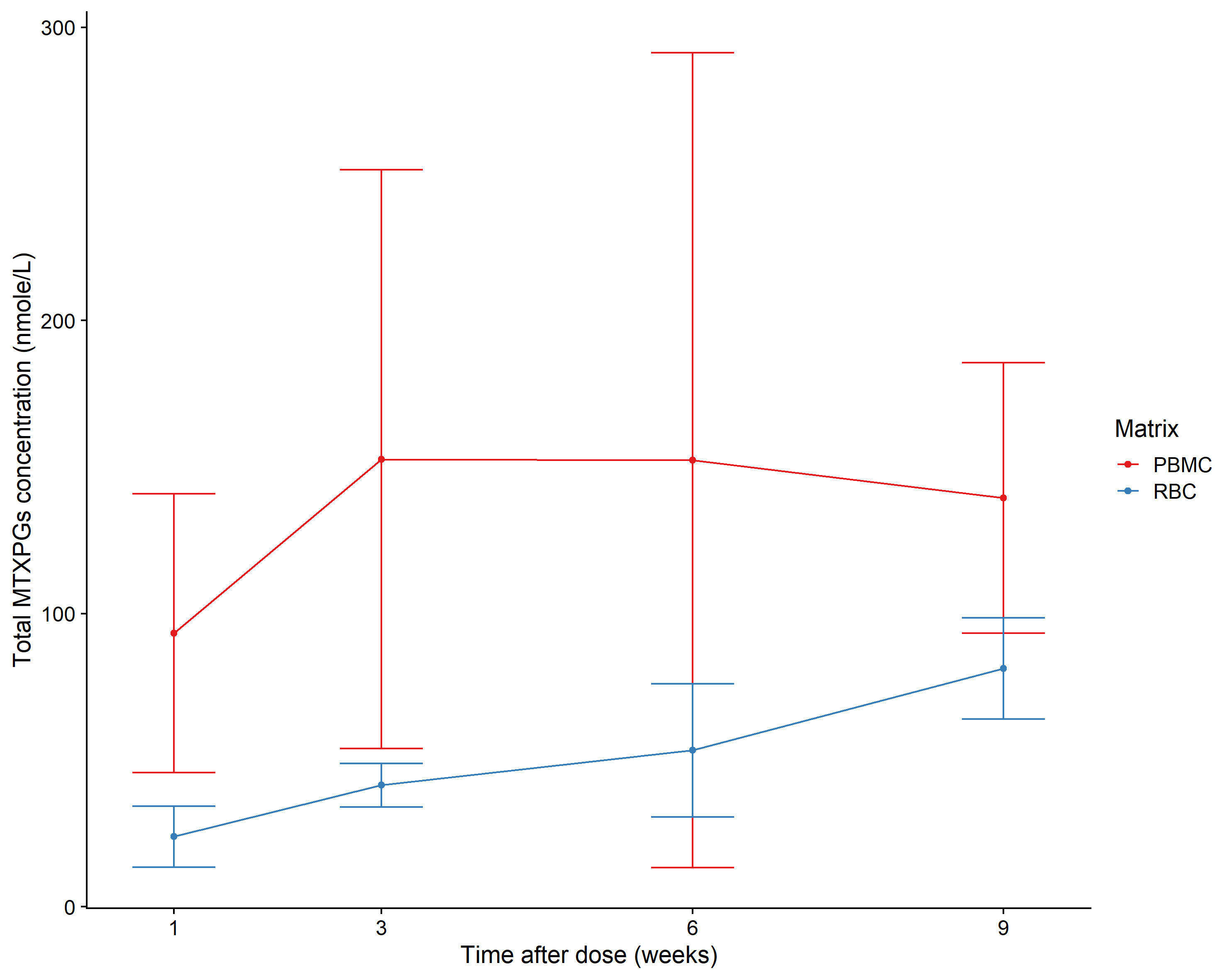


Supplementary figure 2: Mean total MTX-PGs concentrations± standard deviation- extracted from red blood cells versus peripheral blood mononuclear cells

# References:

10. Sonemoto, E., et al., Practical determination of methotrexate in serum of rheumatic patients by LC‐MS/MS. Biomedical Chromatography, 2012. 26(11): p. 1297-1300.

12. den Boer, E., et al., Measuring methotrexate polyglutamates in red blood cells: a new LC-MS/MS-based method. Analytical and bioanalytical chemistry, 2013. 405(5): p. 1673-1681.

13. van Haandel, L., et al., Measurement of methotrexate polyglutamates in human erythrocytes by ion-pair UPLC–MS/MS. Bioanalysis, 2011. 3(24): p. 2783-2796.

14. van Haandel, L., et al., A novel high‐performance liquid chromatography/mass spectrometry method for improved selective and sensitive measurement of methotrexate polyglutamation status in human red blood cells. Rapid Communications in Mass Spectrometry: An International Journal Devoted to the Rapid Dissemination of Up‐to‐the‐Minute Research in Mass Spectrometry, 2009. 23(23): p. 3693-3702.

15. Mo, X., et al., Determination of erythrocyte methotrexate polyglutamates by liquid chromatography/tandem mass spectrometry after low-dose methotrexate therapy in Chinese patients with rheumatoid arthritis. Journal of Chromatography B, 2012. 907: p. 41-48.

18. Hawwa, A.F., et al., A novel dried blood spot-LCMS method for the quantification of methotrexate polyglutamates as a potential marker for methotrexate use in children. PloS one, 2014. 9(2): p. e89908.
